# The Polk Country Fire Rescue screening tool for detecting subarachnoid hemorrhage

**DOI:** 10.1186/s12245-024-00722-1

**Published:** 2024-10-08

**Authors:** Krish Patel, Sanjana Konda, Latha Ganti, Anjali Banerjee, Paul Banerjee

**Affiliations:** 1 Council Rock School District, Newton, PA 18940 USA; 2 Warren Alpert Medical School of Brown University, Providence, RI 02903 USA; 3 Orlando College of Osteopathic Medicine Winter Garden, Winter Garden, FL 34787 USA; 4 University of Georgia, Athens, GA 30602 USA; 5 Polk County Fire Rescue, Bartow, FL 33830 USA

**Keywords:** Subarachnoid hemorrhage, Prehospital stroke, Intracerebral hemorrhage

## Abstract

**Introduction:**

The subarachnoid space in the brain contains crucial blood vessels and cerebrospinal fluid. Aneurysms in these vessels can lead to subarachnoid hemorrhage (SAH), a serious stroke subtype with high morbidity and mortality rates. SAH treatment includes procedures like coiling and clipping, but these are available only at comprehensive stroke centers (CSCs), necessitating urgent diagnosis and transfer to specialized facilities.

**Methods:**

This IRB-approved study was conducted by Polk County Fire Rescue (PCFR) in Florida. PCFR, serving an 850,000-person population, implemented a three-step SAH protocol. The protocol uses both Ottawa SAH criteria and recurring symptoms, such as new-onset seizures and high systolic blood pressure, that were identified by EMS. Acute management included administering labetalol, levetiracetam, and ondansetron.

**Results:**

Of 2175 stroke patients, 80 screened positive for SAH and were eligible for transfer. Patients had a median age of 66, and 33% had an initial systolic BP over 220 mmHg. The interfacility transfer rate dropped from 12.9 to 3.6% after implementing the protocol.

**Conclusion:**

The PCFR protocol’s effectiveness suggests its potential for nationwide implementation. Early SAH recognition and prompt transfer to CSCs reduce complications and improve outcomes. Accurate field diagnosis by EMTs can prevent unnecessary transfers and enhance patient care. Future improvements may include portable diagnostic tools and enhanced EMT training to further improve SAH patients’ pre-hospital care.

## Introduction

The subarachnoid space is the space in the brain between the pia and arachnoid mater. It contains the blood vessels that supply various regions of the brain and the cerebrospinal fluid [1].

Aneurysms are bulges or weakenings in the walls of blood vessels, commonly where the vessels split. These weakenings can eventually cause the aneurysm to burst and bleed into the subarachnoid space, leading to a subarachnoid hemorrhage (SAH), the third most common subtype of stroke [1–3]. Intracerebral hemorrhage comprises 15% of all strokes, of which 54% are subarachnoid hemorrhages. A SAH is often a catastrophic clinical event with significant morbidity and mortality. Approximately 25% of SAH patients die before reaching the hospital, and of the survivors, 50% suffer permanent disability. The 30-day mortality rate for SAH is 50%, and 15% die before reaching the hospital because there is a particular risk of early and devastating re-bleeding [4, 5].

Treatment for SAH can include coiling or clipping for aneurysms, or specific reversal agents for those on anticoagulants. In patients on anticoagulation with a subarachnoid hemorrhage, reversal agents are used to counteract the effects of anticoagulant (blood-thinning) treatment, reducing the risk of future bleeding [6]. An endovascular coiling method involves inserting a catheter into the femoral artery and guiding it to the location of the brain aneurysm. The aneurysm is then sealed off to stop additional bleeding by deploying small platinum coils, forming a blood clot.

Aneurysm clipping, an invasive surgical method, entails opening the skull to access the aneurysm directly. The aneurysm is isolated from the regular blood circulation with a tiny metal clip that a neurosurgeon inserts at its neck to stop it from rupturing or bleeding again [7]. These treatments unfortunately are only available at comprehensive stroke centers (CSCs) and are not provided at all hospitals [6, 7]. Therefore, a patient with SAH has the best outcome at a facility that can provide specific treatment. This necessitates urgent assessment, where the shorter the time from symptom onset to diagnosis and therefore treatment, the better.

There are several types of stroke center designations. Primary stroke centers, or PSCs provide stroke patients with specialized care paths and protocols, and can administer thrombolytics for ischemic stroke and supportive care for intracerebral hemorrhage [8]. Thrombectomy Capable Stroke Centers (TSC) can provide endovascular therapy in addition to tissue plasminogen activators (tPA) for ischemic stroke [9]. A comprehensive stroke center (CSC).

can do all of the above, and provide coiling or clipping of aneurysms in cases of intracerebral hemorrhage. Essentially, a CSC can provide care for all types of stroke patients [8, 9].

Prehospital treatment is crucial and includes transferring the patient to a hospital with neurocritical/neurosurgical expertise after considering breathing, circulation, and airway during triage. Diagnosing subarachnoid hemorrhage can be nuanced [2]. In fact, 33% of patients present no symptoms except for a headache [1]. This makes it particularly challenging for Emergency Medical Services (EMS). Therefore, developing and adhering to protocols is crucial for EMS to be able to accurately identify SAH cases. This takes a great deal of discipline as the traditional teaching is that the presentation of a SAH is a sudden, severe headache, often referred to as the “worst headache of [the patient’s] life” [1].

Over the past few decades, the incidence rate of subarachnoid strokes has declined, which can be attributed to improvements in lifestyle habits, such as lower rates of smoking and methods of controlling hypertension [4]. Additionally, there are country-specific variations in incidence, ranging from a 59% decline in Japan to a 14% decrease in North America. These variations are likely associated with variations in the prevalence of smoking [4].

In response to the lack of sufficient prehospital research and consensus on effectively detecting subarachnoid hemorrhages in the field, Polk County Fire Rescue (PCFR) designed and implemented a three-step SAH protocol. In this paper, the authors describe their experience with using this protocol to identify patients likely to experience SAH, thereby minimizing the risk of treatment delay with interhospital transfers.

## Methods

Located in Polk County, Florida, PCFR is the 39th largest fire department in the country, completing more than 102,000 EMS transports. It serves an 850,000-person population by covering an area of more than 2,010 square miles, responding to more than 125,000 patient calls annually, and treating more than 900 stroke victims. Under the PCFR system, there are two TSCs, three PCPs, and zero CSCs locally, but there are four CSCs in surrounding counties. This necessitates out-of-county transfers, which can mean longer transport times. One of the inherent challenges of EMS transport is taking patients to the right hospital at the right time for the patient to have the best health outcomes. This study is an IRB-approved prospective observational study conducted as part of Polk County’s EMS system’s robust quality and research program.

**Fig. 1:**
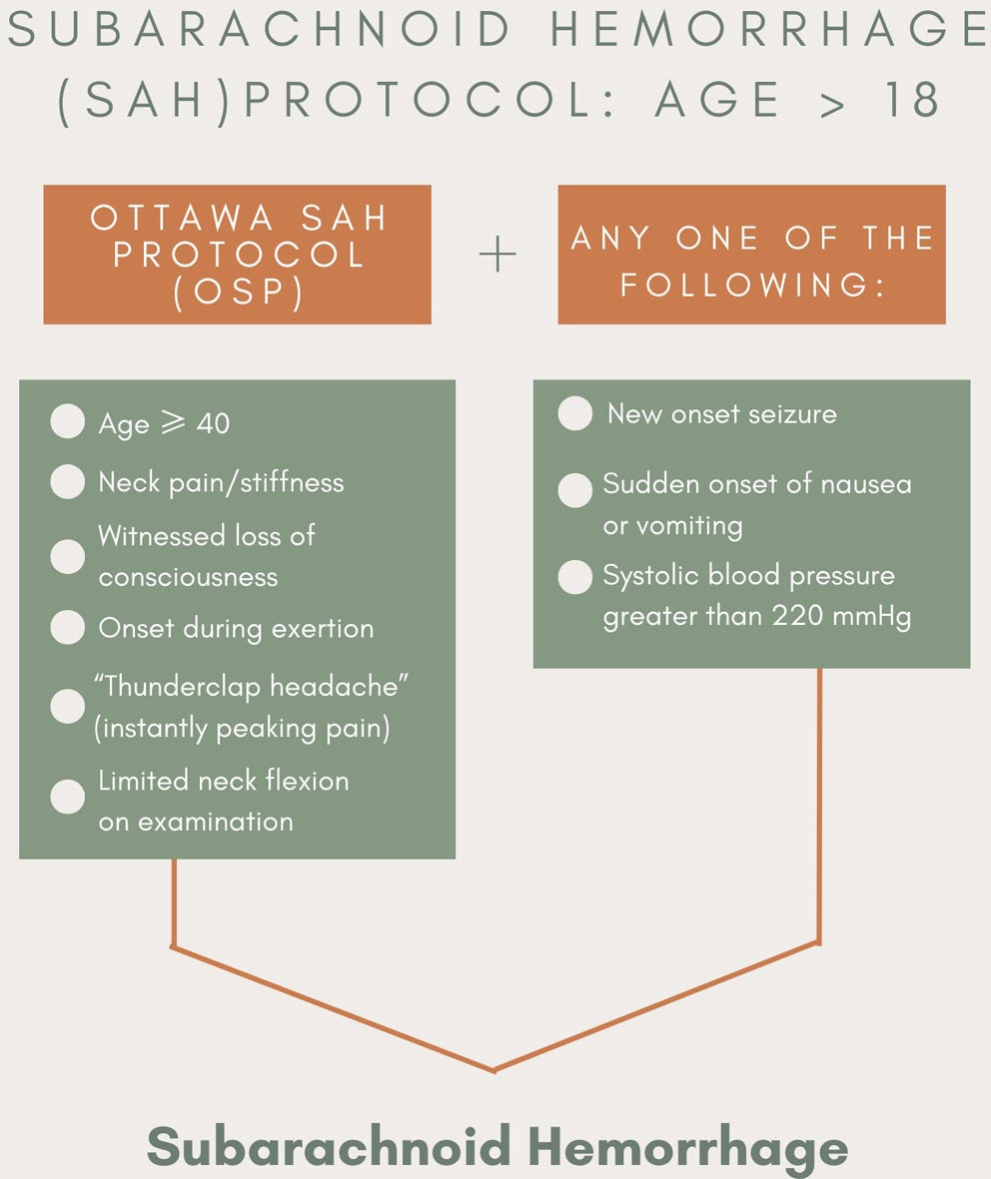
Infographic on specific PCFR protocol used to detect a Subarachnoid Hemorrhage

Our prehospital subarachnoid protocol employs two notable tools that clinicians typically use in the pre-diagnostic and post-diagnosis process. The Ottawa SAH criteria is a rule-out tool for subarachnoid hemorrhage to determine if a patient with a headache needs additional testing, such as a Computed Tomography (CT) scan. The Ottawa SAH rule is a commonly used technique that considers clinical criteria that indicate a high risk for SAH (Fig. 1) [10]. The second criteria used to help detect SAH were based on frequent recurring symptoms that Emergency Medical Technicians (EMTs) noticed in the field and recorded. PCFR deduced three symptoms: new- onset seizure, sudden onset of nausea or vomiting, and elevated systolic blood pressure (greater than 220 mmHg).

Specifically, the protocol employed is applicable for patients 18 years or older who screen positive per the Ottawa SAH rule and have any one of the following symptoms: a new onset seizure, sudden onset of nausea or vomiting, or a systolic blood pressure of > 220 mmHg (Fig. 1). If patients screened positive for SAH by the protocol, acute management was then instituted in the field and consisted of an administration of labetalol 20 mg IV bolus over 2 min or.

**Fig. 2:**
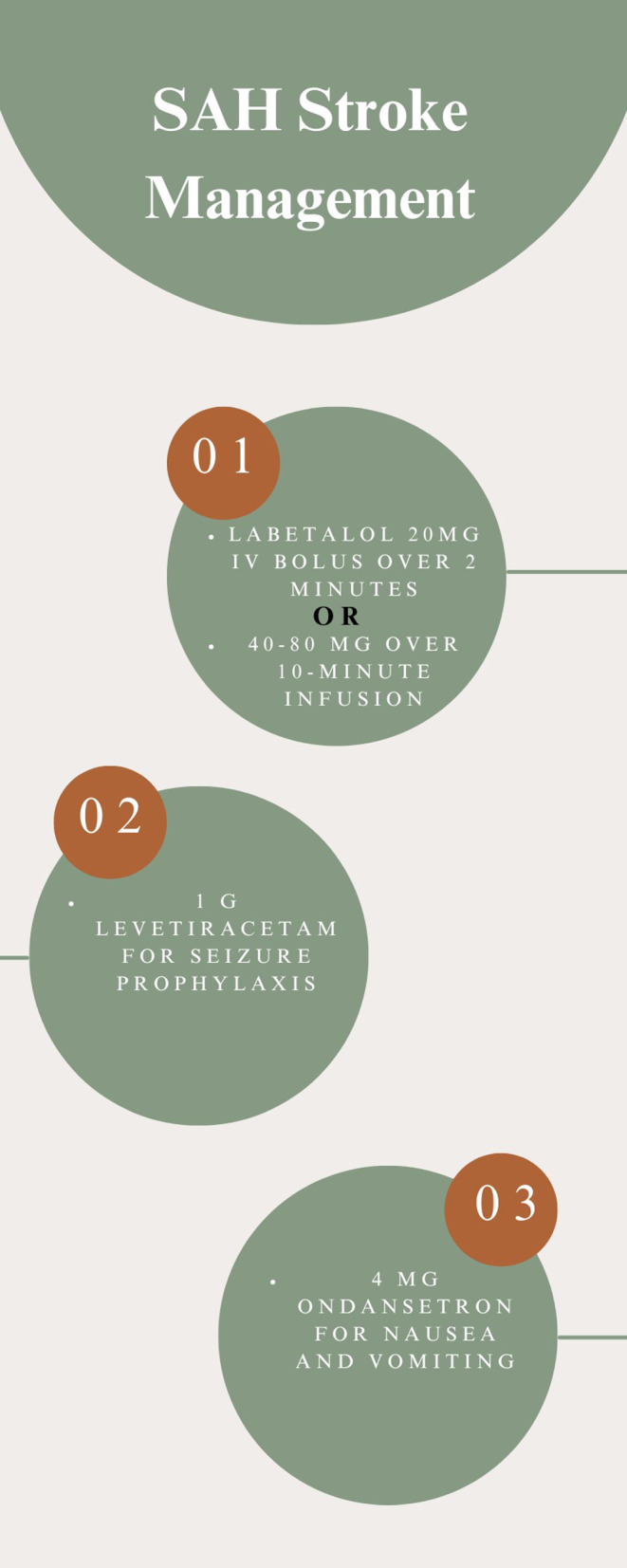
Infographic on specific SAH management post-SAH identification

40–80 mg over 10 min infusion, followed by 1 g levetiracetam for seizure prophylaxis, and 4 mg ondansetron for nausea or vomiting (Fig. 2).

Data collection occurred as part of our PCFR quality registry. The study determination received an exempt determination from our institution (Study #SBE 1713224).

Interhospital transfer rates before and after the introduction of this protocol were examined. Data on transfers for SAH were obtained at each of our hospital partner sites’ monthly quality improvement meetings.

## Results

**Fig. 3:**
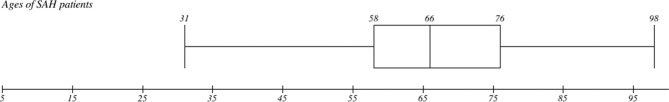
Boxplot of the age spread of SAH patients

A total of 2175 patients were transported for stroke in Polk County. Of this 2022–2023 cohort, 80 (3.68%) cases screened positive for subarachnoid hemorrhages by the protocol and were eligible for out-of-country transfer to CSCs. 42% of the patients were female and 58% were male, with a median age of 66 years (IQR 58–76, range 31–98) (Fig. 3).

**Fig. 4:**
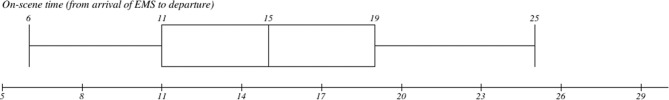
Boxplot of the on-scene time (from arrival of EMS to departure

The median on-scene time, the time from arrival of EMS to departure, was 15 min (IQR 11–19) (Fig. 4).

**Fig. 5:**
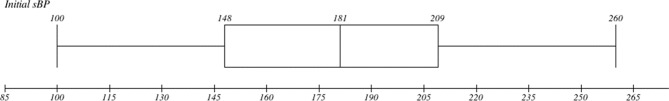
Boxplot of the initial sBP spread of SAH patients

The median initial systolic blood pressure (sBP) was 181 mmHg (IQR 148–209, range 100–260), and 33% of patients had an initial sBP greater than 220 mmHg (Fig. 5).

Before the protocol was implemented, the interfacility transfer rate in Polk County was 12.9%. This rate decreased to 3.6% after it was implemented.

## Discussion

The results of this study demonstrate that the PCFR SAH protocol is effective in decreasing interfacility transport for SAH. Recognizing SAH in the early stages is crucial for stopping bleeding in the intracranial subarachnoid space. Early identification leads to timely transport to a comprehensive stroke center (CSC).

Interfacility transfer for stroke can be associated with significant morbidity and mortality. First, is the delay in treatment which can include anticoagulant reversal, coiling or clipping of aneurysms, or external ventricular drainage. Second, there exist risks of morbidity associated with the transfer process include falls while loading and unloading, EKG leads coming off, and delay in medications to control blood pressure or pain. And finally, there is a true financial burden for the patient, as often there are two hospital bills, as well as the cost of the ambulance transfer.

Numerous studies have been conducted to assess the impact of delays associated with interhospital transfers on outcomes. A study of 107 patients transferred for ICH reported that 30% underwent neurosurgical intervention upon arrival to the CSC, and another 7% had a neurosurgical procedure within 24 h [11].

A 2024 study compared the outcomes of ischemic stroke patients with large vessel occlusions to patients who presented directly to a thrombectomy capable center versus patients who arrived as transfers. This study of almost a thousand patients demonstrated that prolonged transfer times and evolution of ischemic change were associated with worse EVT outcomes [12].

A study within a hub-and-spoke telestroke network reported that a patient’s chance of having thrombectomy decreased by 1% for each additional minute of transfer time over 60 min, with the most pronounced transfer delays occurring at night [13]. The delay of treatment when a patient arrives during night shift is corroborated by another study that examined door to needle times for ischemic strokes based on which shift the patient arrived [14].

A retrospective chart review of ischemic and hemorrhagic strokes, including SAH and transient ischemic attacks, found that recognition of stroke by EMS providers was independently associated with faster door-to-physician time, faster door-to-CT time, and greater odds of receiving specific treatment. These results were independent of age, NIHSS, symptom duration, and EMS prenotification [15].

A study of over 36,000 patients with intracerebral hemorrhage reported that patients were more likely to receive neurosurgical intervention and be alive at 90 days when admitted to comprehensive stroke centers versus other hospitals [16, 17].

## Limitations and future directions

The data reviewed here demonstrate that the interfacility transfer rate was significantly lower with implementation of the PCFR SAH protocol. While one can surmise that this metric translates to improved morbidity and mortality, the current study does not report specific outcomes. The authors are currently collecting specific measures to further refine the utility of the protocol.

## Conclusions

The Polk County Fire Rescue (PCFR) protocol for subarachnoid hemorrhage is a useful tool to reduce interfacility transfers for patients with SAH.

## Data Availability

No datasets were generated or analysed during the current study.
